# Scientific landscape of oxidative stress in sarcopenia: from bibliometric analysis to hotspots review

**DOI:** 10.3389/fmed.2024.1472413

**Published:** 2024-11-11

**Authors:** Linjie Wang, Dongliang Guo, Yi Huang, Pan Long, Xin Zhang, Ling Bai, Jiancheng Liu, Xiaomin Hu, Rizhao Pang, Xiang Gou

**Affiliations:** ^1^Department of Rehabilitation Medicine, The General Hospital of Western Theater Command, Sichuan, Chengdu, China; ^2^Sichuan Clinical Medical Research Center for Traditional Chinese Medicine Orthopedics and Sports Medicine Rehabilitation, Sichuan, Chengdu, China; ^3^Department of Ophthalmology, The General Hospital of Western Theater Command, Sichuan, Chengdu, China

**Keywords:** bibliometric analysis, bibliometric, CiteSpace, oxidative stress, sarcopenia

## Abstract

**Objective:**

Sarcopenia is a significant healthcare challenge in the aging population. Oxidative stress (OS) is acknowledged to play a pivotal role in the pathological progression of sarcopenia. Numerous studies have demonstrated that mitigating or eliminating OS can ameliorate the pathological manifestations associated with sarcopenia. However, current clinical antioxidant therapies often fall short of anticipated outcomes. This bibliometric analysis aims to delineate prevailing research trends, thematic emphases, focal points, and developmental trajectories within the domain of OS in sarcopenia, while also endeavoring to explore prospective anti-oxidative stress strategies for future clinical interventions.

**Methods:**

Relevant publications were retrieved from the Web of Science (WOS) Core Collection database for the period 2000-2024. Citespace was employed for retrieving and analyzing trends and emerging topics.

**Results:**

In the field of OS in sarcopenia, the number of publications has significantly increased from 2000 to 2024. The United States and China are the primary contributors to global publication output. The most productive research institution is INRAE. The most prolific author is Holly Van Remmen from the United States, while the most frequently cited author is Cruz-Jentoft AJ from Spain. Experimental Gerontology is the journal with the highest volume of published articles, whereas the Journal of Gerontology Series A: Biological Sciences and Medical Sciences holds the record for the highest number of citations. The research keywords in this field can be categorized into eight domains: “Physiology and anatomy”, “Physiological mechanisms”, “Pathology associations”, “Experimental studies”, “Nutrition and metabolism”, “Sports and physical activities”, “Age” and “Oxidation and antioxidation”. Moreover, recent years have seen the emergence of “TNF-*α*,” “insulin resistance”, “mitochondrial autophagy”, “signal pathways”, and “mechanisms” as focal points in the realm of OS in sarcopenia, encompassing related fundamental research and clinical translation.

**Conclusion:**

This bibliometric and visualization provides a comprehensive analysis of the global research landscape in the field of OS in sarcopenia, identifies priorities, summarizes the current research status and suggests possible future research priorities. In addition, in order to benefit more sarcopenia patients, strengthening cooperation and communication between institutions and research teams is the key to the future development of this field. Given the expectation that research on OS in sarcopenia will remain a prominent area of interest in the future, this article could serve as a valuable resource for scholars seeking to shape future studies through an understanding of influential scholarly contributions and key research findings.

**Systematic review registration:**

https://www.crd.york.ac.uk, identifier CRD42024528628.

## 1 Introduction

Sarcopenia, marked by the rapid decline in muscle mass and function, is an advancing and prevalent skeletal muscle condition (1). In clinical practice, it often manifests insidiously, significantly increasing elderly patients’ susceptibility to falls, fractures, functional decline, and mortality (1–3). Data released by the Asian Working Group for Sarcopenia (AWGS) indicate that sarcopenia’s prevalence among elderly individuals across Asia ranges from 5.5 to 25.7% (2). The incidence of sarcopenia increases with age. Research indicates that sarcopenia’s prevalence can reach up to 15% among individuals aged 65 years and above, and up to 50% among those aged 80 years and above (4). A 2019 study conducted by the University of Oxford in the United Kingdom and the Abbott-Nutrition Division in the United States revealed that patients hospitalized due to sarcopenia incurred average annual medical costs as high as $2,376 and £2,707 (5, 6). The acceleration of global population aging is anticipated to lead to a projected increase in worldwide sarcopenia cases to 500 million by 2050, consequently resulting in a substantial rise in both family economic and social medical burdens (7).

Reactive oxygen species (ROS), which include molecules such as hydrogen peroxide, superoxide anion, and hydroxyl radical are known for their high reactivity and oxygen content (8). These highly reactive molecules exhibit strong oxidizing abilities and play important roles in biological systems. Excessive production of ROS can overwhelm the body’s natural antioxidant defenses, resulting in oxidative stress (OS) closely associated with the pathological advancement of sarcopenia (9). OS can disrupt the equilibrium of skeletal muscle hypertrophy and regeneration through mechanisms such as disturbances in protein synthesis and catabolism, impairment of neuromuscular junction function, and inhibition of satellite cell activation, proliferation, and myoblast differentiation (10–12). Furthermore, inflammation and mitochondrial dysfunction intensify OS, creating a vicious cycle that promotes muscle cell death and progressively reduces muscle fiber number and size (13).

**Figure 1:**
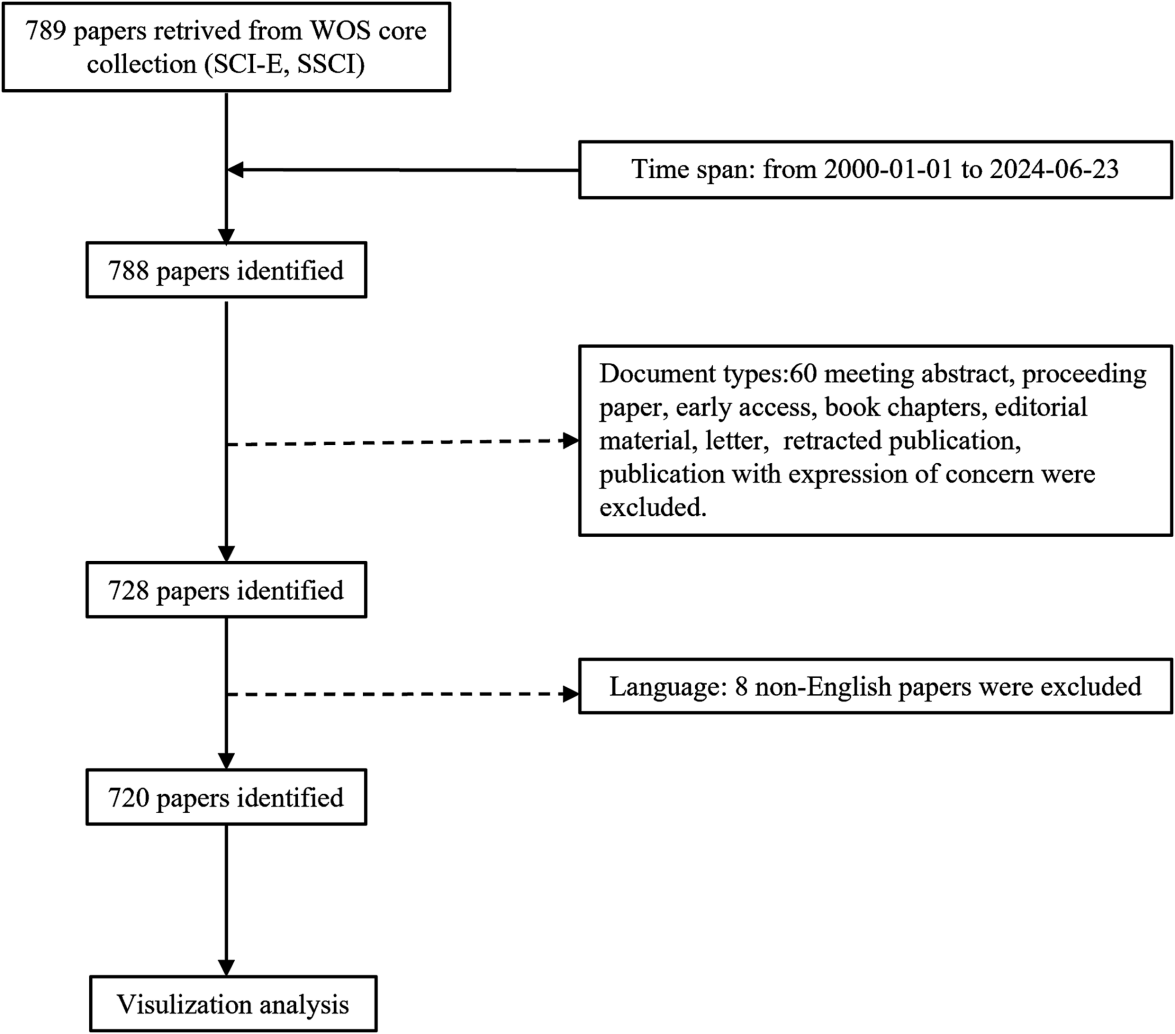
studies inclusion.

Owing to the scarcity of bibliometric studies on research trends related to OS in sarcopenia, a combination of bibliometric analysis and comprehensive review is employed in this paper to furnish researchers with a foundational knowledge base and outline potential directions for future research. Consequently, an extensive search for articles pertaining to OS and sarcopenia since the 21st century was conducted, significant articles in this field were explored (Figure 1; Supplementary Table S1), and the current research status and cutting-edge trends were analyzed. The objectives were: (1) to identify the predominant academic groups in the domain of OS research in sarcopenia; (2) to conduct a comprehensive analysis of the research foundation, present highlights, and future research directions; (3) based on literature metrology study results, to review key areas and discuss the specific role of crosstalk between oxidative stress and complex pathological mechanisms in muscle diseases.

## 2 Materials and methods

### 2.1 Inclusion and exclusion criteria

The Web of Science (WOS) core collection database is widely acknowledged as a comprehensive, systematic, and authoritative repository of influential academic journals, extensively utilized for bibliometric analysis (14, 15). Relevant literature was consulted, and search strategies pertaining to OS and sarcopenia were formulated (Supplementary Table S1) (16, 17). The inclusion and exclusion criteria, as depicted in Figure 1, were: (i) the study period from January 1, 2000, to June 23, 2024; (ii) inclusion of articles and reviews only; and (iii) restriction to publications in English. A total of 720 articles were yielded by the search. Each publication included titles, keywords, publication dates, journal names, author information, countries of origin, affiliated institutions, and citation counts. The literature search and evaluation were independently conducted by two researchers, who resolved any discrepancies through discussion with a third party.

### 2.2 Tools and data analysis

To conduct further analysis, the plain text export of the acquired articles as “download XXX.txt” was imported into CiteSpace 6.3.1, along with their complete data and references. During the process of mapping and visualizing knowledge figures, a set of primary steps including temporal segmentation, thresholding, modeling, pruning, merging, and mapping was strictly adhered to. CiteSpace encompasses several fundamental concepts such as burst detection, betweenness centrality, and heterogeneous networks, which contribute to the timely visualization of research status updates and the identification of prominent areas and frontiers.

Nodes in different maps show authors, institutions, countries, or keywords, with links between nodes describing co-occurrence or co-citation. Node size represents the frequency of occurrence or citation, while node color denotes the respective years of occurrence or citation. A thicker link denotes more frequent coexistence or cooperation between the nodes. It is noteworthy that betweenness centrality is a tool used to assess the significance of each element in the visualization network. Typically, elements with a betweenness centrality of less than 0.1 will have a purple outer circle, signifying that the node is a hub with significant interactive importance. A modularity value (Q-value) >0.3, combined with an average contour value (S-value) >0.7, suggests that clustering is both statistically significant and reasonable (18). Furthermore, burst detection, pioneered by Kleinberg, is a potent analytical tool. Its strength is measured by the growth rate of keyword citations over time. Therefore, it can be used to investigate research hotspots or frontiers over a specific period, illustrating their evolution (19).

## 3 Results

### 3.1 The publication outputs trend

**Figure 2:**
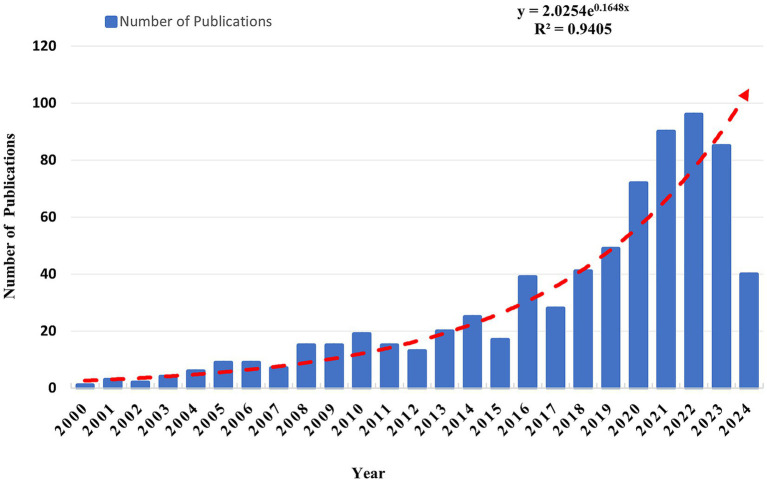
Trends of publications on OS in sarcopenia (the formulae only utilize publications from 2000 to 2023).

The annual publication production has steadily increased from January 1, 2000, to December 31, 2023, as evidenced by the data (Figure 2). Additionally, based on the fitting curve from 2000 to 2023 (R^2^ = 0.9405), the projected trend indicates a future increase. This suggests that OS in sarcopenia is a hot topic in the field, attracting more researchers to contribute. Significant progress is projected in understanding the molecular mechanisms related to OS in sarcopenia, potentially providing new ideas for treatment and interventions.

### 3.2 Distribution of countries/regions and institutions

By statistically analyzing the number of papers published by countries, regions, institutions, and authors, the key countries, research institutions, and authors with strong influence in the research field of OS in sarcopenia can be identified, and the cooperation relationships between them can be clarified.

**Figure 3:**
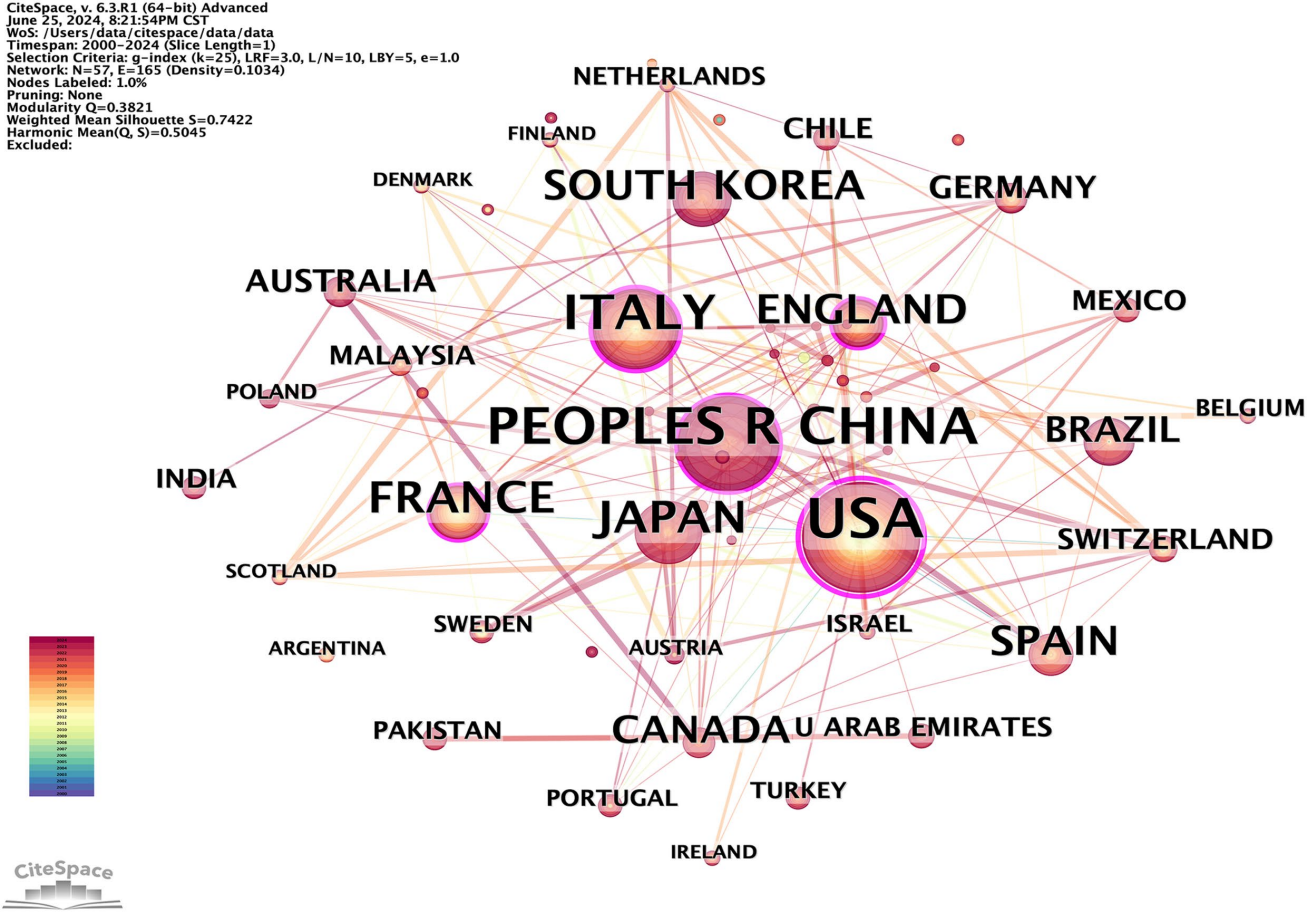
CiteSpace visualization map of publications on OS in sarcopenia from different countries/regions.

**Figure 4:**
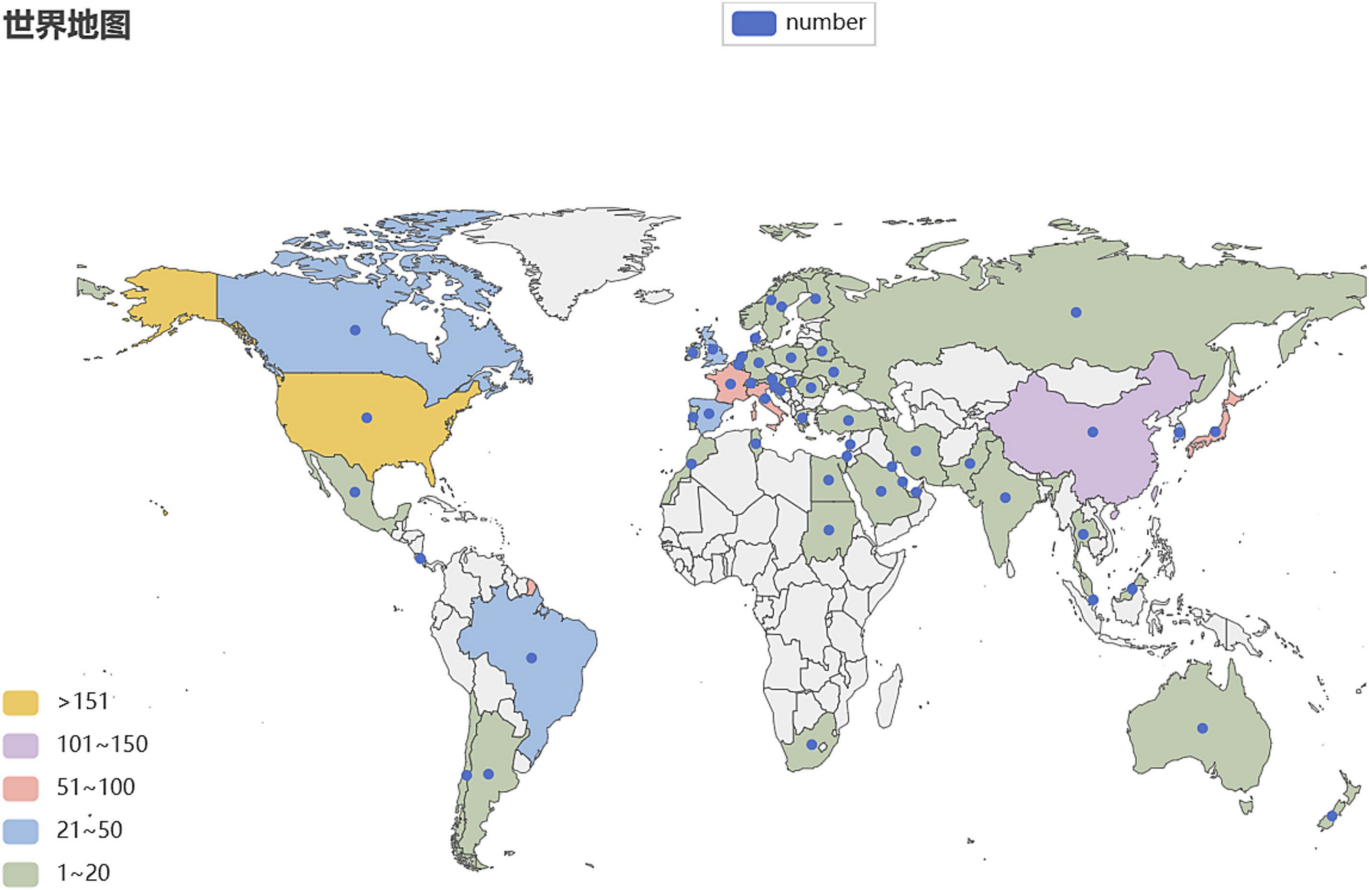
The distribution of countries by OS in sarcopenia publishing volume.

To identify the leading countries/regions in this field, a bibliometric analysis of publications from various countries/regions was conducted using CiteSpace. Publications on OS in sarcopenia originate from a total of 57 nations/regions. There are 57 nodes and 165 links representing the collaborative relationships between nations and regions (Figure 3). This indicates that there is a certain basis of cooperation between countries/regions. The world map generated by Python 3.8, shown in Figure 4, was utilized to visualize the number of publications contributed by each country/region, with each country/region represented by a distinct color. Publications and centrality were used to rank the top 10 nations in Table 1. The United States, China, and Italy were the most prolific, accounting for 26.11, 17.36, and 13.47% of all publications, respectively. Japan, France, South Korea, the United Kingdom, Spain, Canada, and Brazil followed in the rankings. It is worth noting that among the top 10 countries in the number of publications, eight countries, including the United States, Italy, and France, are also among the top 10 in intermediary centrality.

**Table 1:** Top 10 countries and regions by publications and centricity.

Publications			Centrality		
Ranking	Country/region	Frequency	Ranking	Country/region	Frequency
1	USA	188 (26.11%)	1	USA	0.63
2	Peoples R China	125 (17.36%)	2	Italy	0.3
3	Italy	97 (13.47%)	3	France	0.15
4	Japan	64 (8.89%)	4	England	0.12
5	France	58 (8.06%)	5	Peoples R China	0.11
6	South Korea	44 (6.11%)	6	Canada	0.08
7	England	41 (5.69%)	7	Spain	0.07
8	Spain	41 (5.69%)	8	South Korea	0.06
9	Canada	30 (4.17%)	9	Switzerland	0.06
10	Brazil	25 (3.47%)	10	U Arab Emirates	0.06

**Figure 5:**
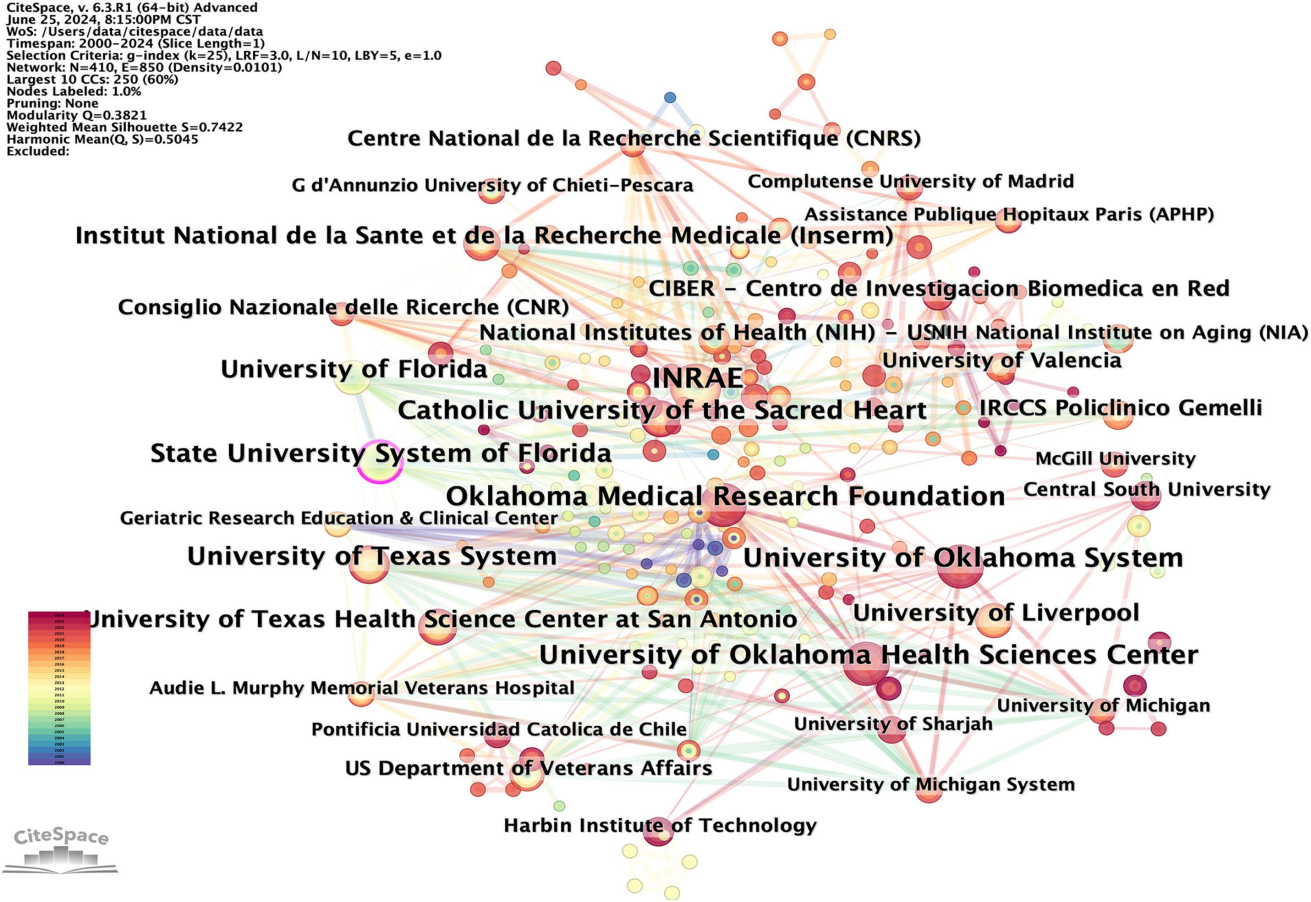
CiteSpace visualization map of publications on OS in sarcopenia from different institutions.

The CiteSpace tool was used to construct a visual mapping, aiming to enhance the clarity of institutions involved in cooperation within the field. A total of 410 institutions have published works in this field (Figure 5). These publications are concentrated in universities worldwide and reflect close cooperative relationships. According to their publications and centrality, the top 10 institutions are presented in Table 2. INRAE (26), Oklahoma Medical Research Foundation (21), University of Oklahoma System (21), University of Oklahoma Health Sciences Center (21), and University of Texas System (20) are the top five institutions contributing the most to this discipline. State University System of Florida (0.1), University of Florida (0.1), Aalborg University (0.09), University of Texas System (0.08), and Consiglio Nazionale delle Ricerche (0.08) round out the top five institutions for centrality. Consistent with the main research countries, the leading research institutions in this field are primarily in European and American countries. This information can be utilized by experts in this field to inform research, study, and further cooperation.

**Table 2:** Publications and centricity top 10 institutions.

Publications			Centrality		
Ranking	Institution	Frequency	Ranking	Institution	Frequency
1	National Research Institute for Agriculture, Food and Environment (INRAE)	26	1	State University System of Florida	0.1
2	Oklahoma Medical Research Foundation	21	2	University of Florida	0.1
3	University of Oklahoma System	21	3	Institut National de la Sante et de la Recherche Medicale (Inserm)	0.09
4	University of Oklahoma Health Sciences Center	21	4	University of Texas System	0.08
5	University of Texas System	20	5	Consiglio Nazionale delle Ricerche	0.08
6	Catholic University of the Sacred Heart	18	6	University of Texas Health Science Center at San Antonio	0.07
7	State University System of Florida	18	7	University of Padua	0.07
8	University of Florida	17	8	Complutense University of Madrid	0.06
9	Institut National de la Sante et de la Recherche Medicale (Inserm)	15	9	University of California System	0.05
10	University of Liverpool	14	10	Buck Institute for Research on Aging	0.05

### 3.3 Authors and co-cited authors

**Figure 6:**
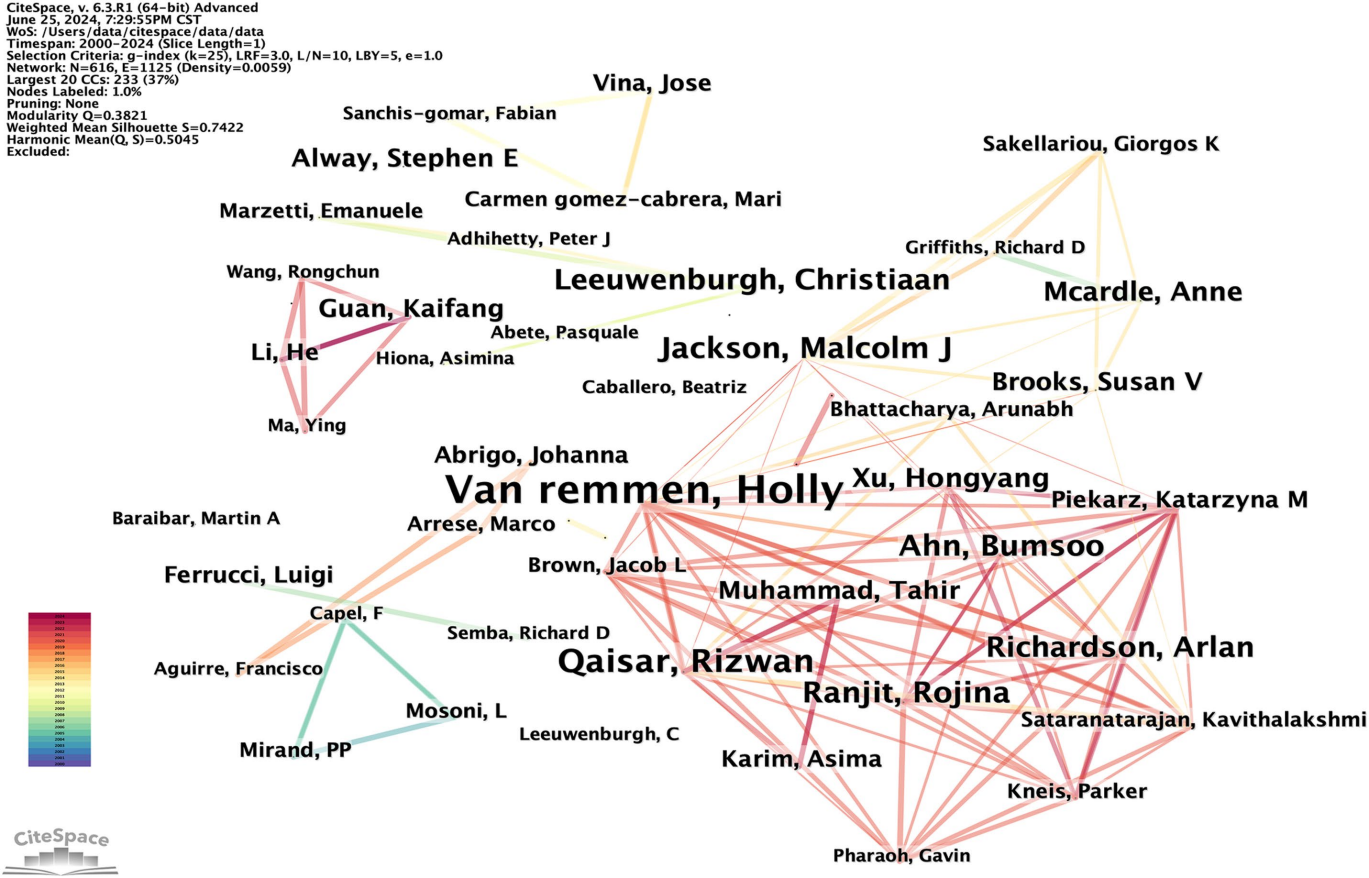
CiteSpace visualization author co-occurrence map of OS in sarcopenia.

**Figure 7:**
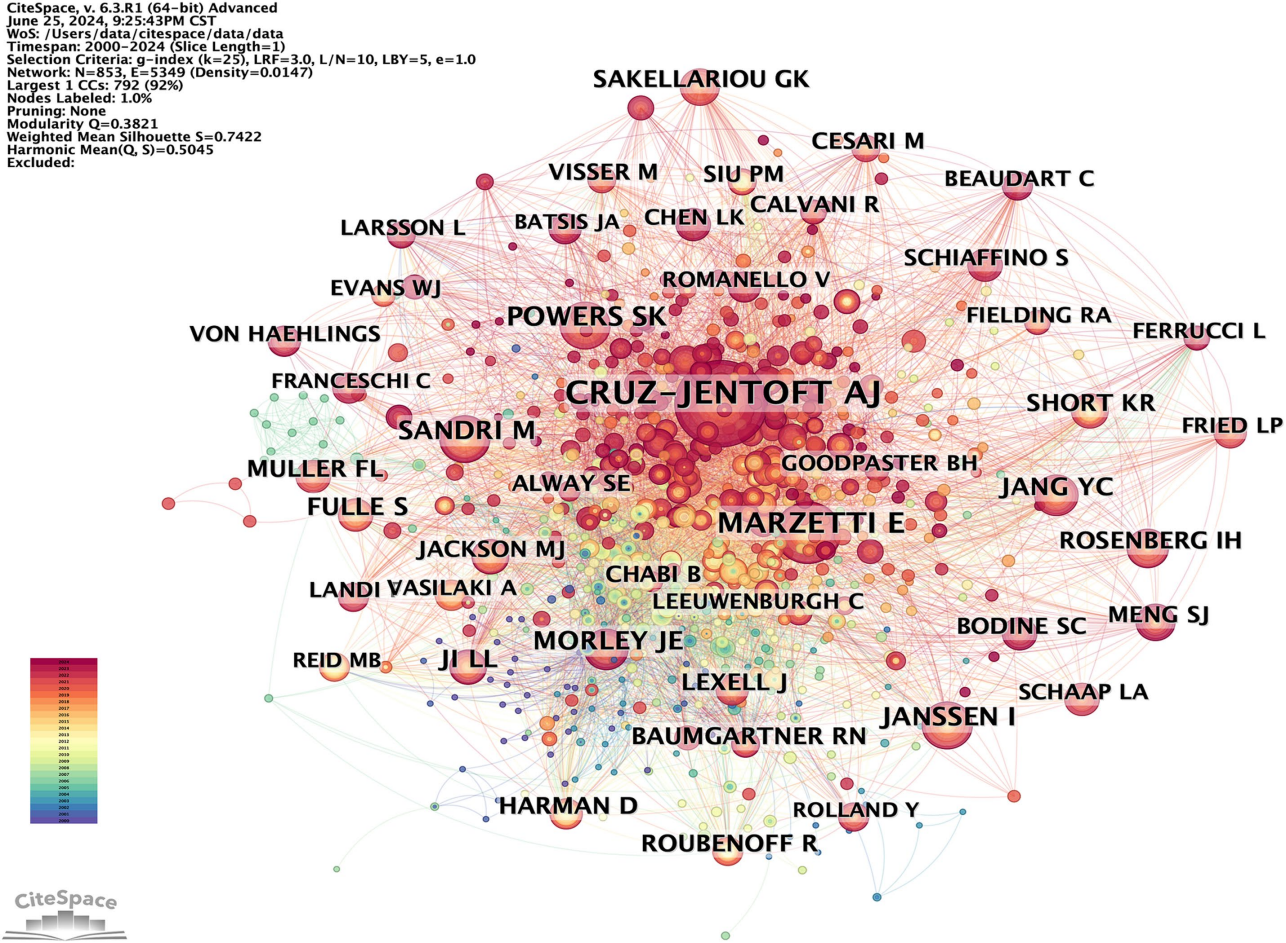
CiteSpace visualization author co-citation map of OS in sarcopenia.

Research typically requires collaboration among multiple researchers. By analyzing the characteristics of the author co-occurrence network, the central figures in a given research field can be identified based on their level of cooperation with other authors. The author co-occurrence network depicted in Figure 6 consists of 616 nodes and 1,125 links, with a density of 0.0059. This indicates a high level of collaboration among scholars in the field, with some potentially establishing formal research groups. A total of 616 authors have conducted relevant research. The most prolific author is Holly Van Remmen from the United States, followed by Rizwan Qaisar from the United Arab Emirates and Malcolm J. Jackson from the UK. Co-citation occurs when two authors’ works are cited together by a third author’s work. This indicates a close academic relationship and suggests their proximity within the scholarly domain (20). The author co-citation network depicted in Figure 7 consists of 853 nodes and 5,349 links, with a density of 0.0147. The top three most frequently co-cited authors are Cruz-Jentoft AJ from Spain, Marzetti E from Italy, and Powers SK from the United States. Table 3 lists the top 10 high-yield authors and the top 10 most cited authors.

**Table 3:** Top 10 author by publication and co-citation.

Author’s publication			Co-cited author		
Ranking	Author	Frequency	Ranking	Author	Frequency
1	Van remmen, Holly	23	1	Cruz-Jentoft AJ	247
2	Qaisar, Rizwan	12	2	Marzetti E	144
3	Jackson, Malcolm J	9	3	Powers SK	109
4	Ranjit, Rojina	9	4	Morley JE	90
5	Ahn, Bumsoo	9	5	Janssen I	81
6	Richardson, Arlan	8	6	Sandri M	80
7	Leeuwenburgh, Christiaan	8	7	Fulle S	72
8	Xu, Hongyang	7	8	Jang YC	72
9	Mcardle, Anne	7	9	Ji LL	69
10	Brooks, Susan V	6	10	Muller FL	57

### 3.4 Journals and cited journals

**Figure 8:**
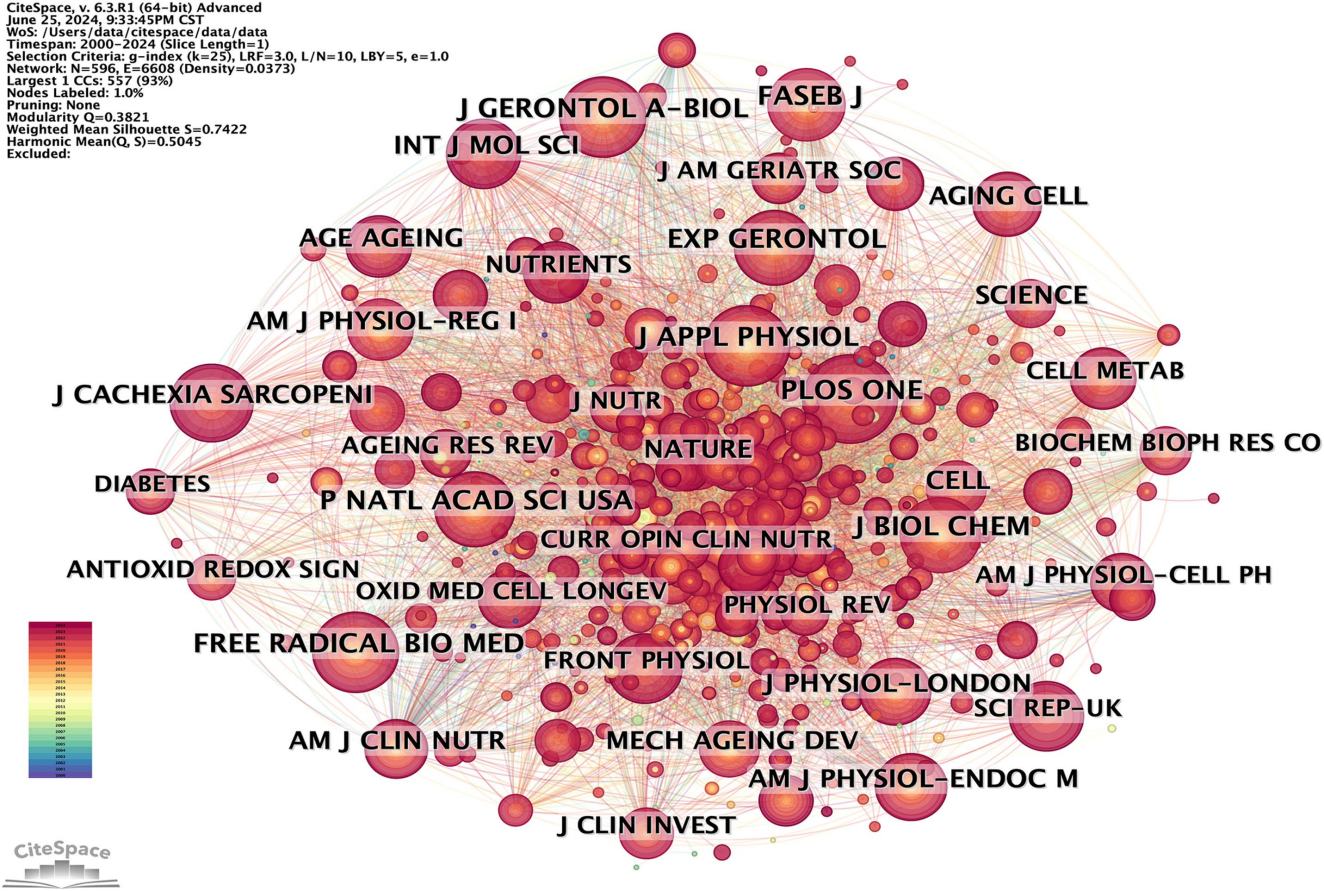
CiteSpace visualization cited journals map of OS in sarcopenia.

Publications on oxidative stress (OS) in sarcopenia were distributed across 596 academic journals (Figure 8). Among these, 219 articles, comprising 30.41% of the total publications, appeared in the top 10 most active journals (Table 4).

**Table 4:** Top 10 journals by publication and citation.

Items	Ranking	Name	Counts	IF(2023)	JCR division
Journals	1	Experimental Gerontology	33	3.3	Q2
	2	International Journal of Molecular Sciences	33	4.9	Q1
	3	Antioxidants	30	6.0	Q1
	4	Journal of Cachexia Sarcopenia and Muscle	25	9.4	Q1
	5	Nutrients	25	4.8	Q1
	6	Free Radical Biology and Medicine	17	7.1	Q1
	7	Aging US	15	3.9	Q2
	8	Journals Of Gerontology Series A-biological Sciences And Medical Sciences	15	4.3	Q1
	9	Aging Cell	13	8.0	Q1
	10	Frontiers in Physiology	13	3.2	Q2
Cited journals	1	Journals Of Gerontology Series A-biological Sciences And Medical Sciences	422	4.3	Q1
	2	Plos One	416	2.9	Q1
	3	Free Radical Biology and Medicine	407	7.1	Q1
	4	Experimental Gerontology	402	3.3	Q2
	5	Journal of Applied Physiology	392	3.3	Q1
	6	Proceedings Of The National Academy Of Sciences Of The United States Of America	381	9.4	Q1
	7	Journal Of Biological Chemistry	364	4.0	Q2
	8	Faseb Journal	344	4.4	Q2
	9	Journal of Cachexia Sarcopenia and Muscle	303	9.4	Q1
	10	Journal Of Physiology-london	299	4.7	Q1

According to Brookes’ Bradford Law, the top 10 journals with the highest publication frequency are acknowledged as pivotal journals in their specific fields (21). These journals serve as the primary options for researchers to submit and refer to articles on OS in sarcopenia. The journals with the highest number of publications were Experimental Gerontology (33) and International Journal of Molecular Sciences (33), followed by Antioxidants (30). The most cited journals were Journals of Gerontology Series A: Biological Sciences and Medical Sciences (422), followed by PLoS One (416) and Free Radical Biology and Medicine (407).

### 3.5 Co-cited references and research clusters

**Figure 9:**
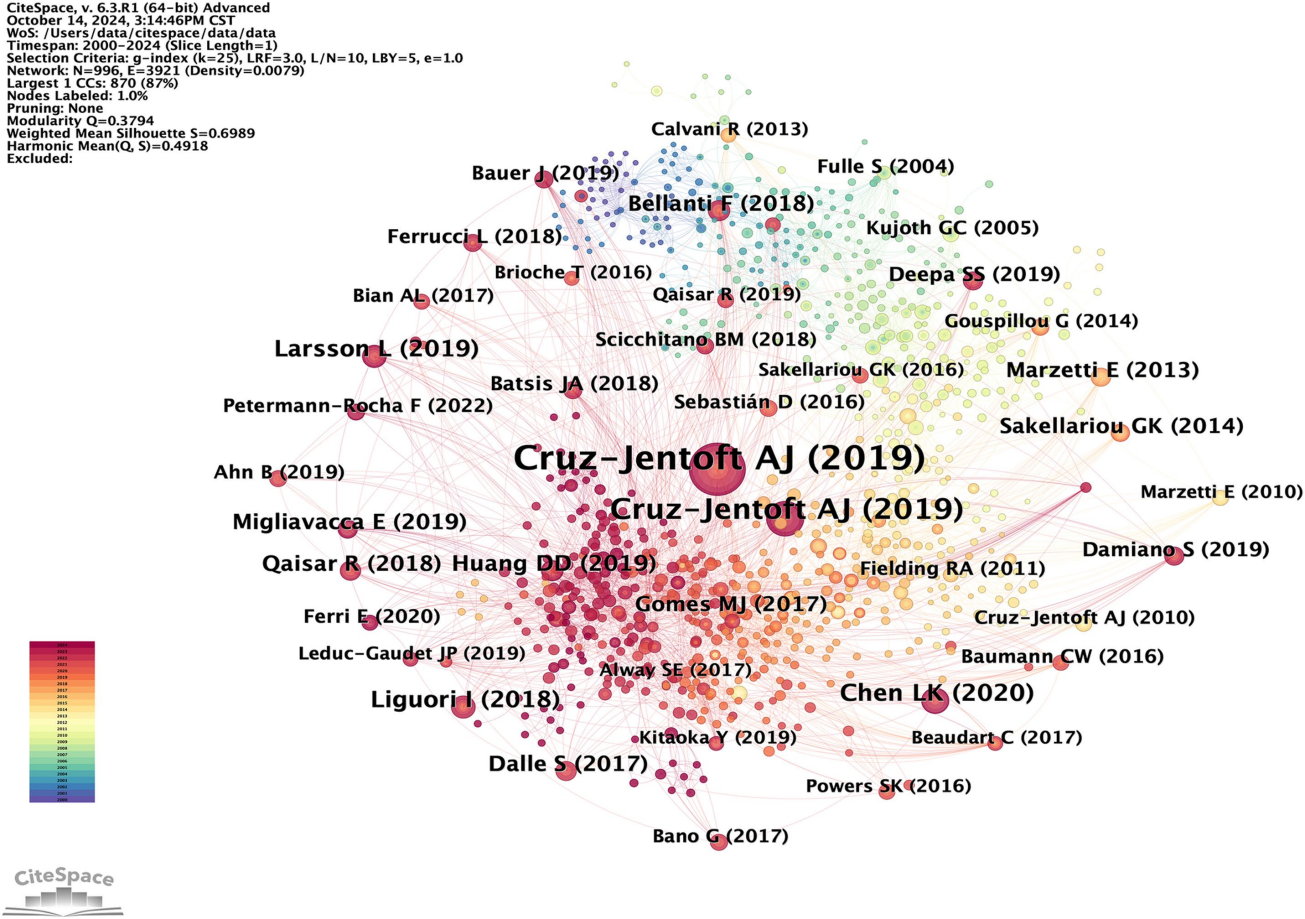
CiteSpace visualization cited literatures map of OS in sarcopenia.

Highly cited papers are authoritative and influential within a specific field, offering researchers a distinctive methodological or theoretical foundation. Reference co-citation analysis is an effective method for evaluating progress and tracing the development of any research field (22). These co-cited papers likely share common characteristics, such as elucidating and supplementing existing theories or methodologies, thereby establishing the groundwork for a specific field. Networks are consequently utilized to delineate research foundations and forecast forthcoming advancements in areas of interest, capturing the dynamic evolution within the knowledge graph. A co-citation network comprising 992 nodes and 3,916 edges was subsequently established (Figure 9). Although there are two nodes in Figure 9 that are both Cruz-Jentoft AJ (2019), we find that their sizes are different. In the cited map, the size of the node represents the cited frequency of the literature. The larger the node, the more frequently the literature is cited. In combination with Table 5, the large Cruz-Jentoft AJ (2019) node and the small Cruz-Jentoft AJ (2019) node are two papers published by Professor Cruz-Jentoft AJ’s team, respectively, in Age and Ageing and Lancet in 2019. They were also the two most frequently cited articles.

**Table 5:** Top 10 literature by high citation.

Title	Journal	Author	Counts	Centrality	Year	Type	Conclusion
Sarcopenia: revised European consensus on definition and diagnosis	Age and Ageing	Alfonso J Cruz-Jentoft et al.	102	0.05	2019	Expert consensus	The European Working Group on Sarcopenia in Older People has updated the expert consensus, aimed at improving understanding to reduce muscle disease and its associated risks.
Sarcopenia	Lancet	Alfonso J Cruz-Jentoft and Avan A Sayer	57	0.04	2019	Review	The definition, screening, diagnosis, differential diagnosis, epidemiology, and pathophysiological mechanisms of sarcopenia, as well as treatment methods (including both drug and non-drug), are summarized.
Asian Working Group for Sarcopenia: 2019 Consensus Update on Sarcopenia Diagnosis and Treatment	Journal of the American Medical Directors Association	Liang-Kung Chen et al.	25	0.01	2020	Expert consensus	The Asian Working Group for Sarcopenia has updated the latest algorithms, protocols, and criteria for the diagnosis of sarcopenia, introduced the concept of “possible sarcopenia,” and summarized the treatment and intervention methods.
Oxidative stress, aging, and diseases	Clinical Interventions in Aging	Ilaria Liguori et al.	22	0.02	2018	Review	The pathophysiology of oxidative stress, its relationship with aging, and its association with age-related diseases have been reviewed. Oxidative stress biomarkers and treatments have also been summarized.
Sarcopenia: Aging-Related Loss of Muscle Mass and Function	Physiological Reviews	Lars Larsson et al.	22	0.06	2019	Review	This review mainly introduces the experimental model, how aging affects different parts of motor units, potential mechanisms, intervention strategies, and specific neuromuscular diseases associated with aging.
Nrf2 deficiency exacerbates frailty and sarcopenia by impairing skeletal muscle mitochondrial biogenesis and dynamics in an age-dependent manner	Experimental Gerontology	Dong-Dong Huang et al.	20	0.04	2019	Original research	The role of Nrf2 in the development of frailty and sarcopenia during aging has been elucidated.
Oxidative stress is increased in sarcopenia and associated with cardiovascular disease risk in sarcopenic obesity	Maturitas	Original research *et al*	18	0.02	2018	Original research	The heightened markers of the oxidative stress cycle in sarcopenia have been revealed, which are associated with an increased risk of cardiovascular disease (CVD) due to obesity.
Oxidative stress-induced dysregulation of excitation-contraction coupling contributes to muscle weakness	Journal of Cachexia, Sarcopenia and Muscle	Rizwan Qaisar et al.	17	0.03	2018	Original research	The dysregulation of excitation-contraction coupling induced by oxidative stress has been shown to lead to muscle weakness.
The Role of Inflammation in Age-Related Sarcopenia	Frontiers in Physiology	Sebastiaan Dalle, Sebastiaan Dalle and Katrien Koppo	17	0.01	2017	Review	The molecular interactions among inflammation, anabolic sensitivity, and muscle protein metabolism in elderly sarcopenia have been summarized.
Mitochondrial oxidative capacity and NAD biosynthesis are reduced in human sarcopenia across ethnicities ^+^	Nature Communications	Eugenia Migliavacca et al.	17	0.01	2019	Original research	The fundamental role of mitochondrial metabolic alterations in the pathological decline of skeletal muscle mass and function among elderly individuals has been demonstrated in the present study.

**Figure 10:**
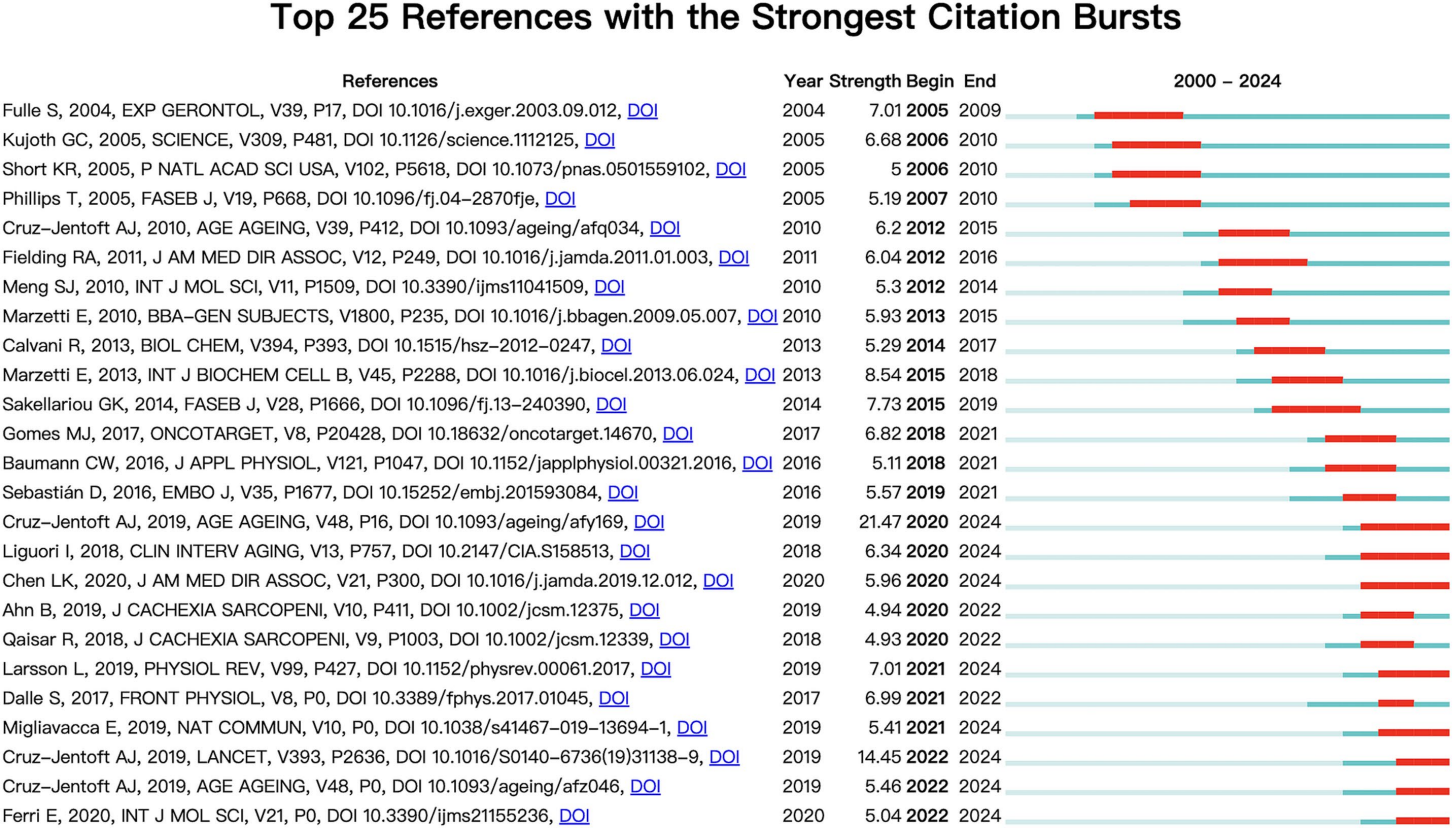
Cited literatures with the strongest citation burst.

The first and third most frequently cited papers were the expert consensus on diagnosis and treatment of sarcopenia formed by European and Asian experts, respectively. The content addresses European and Asian experts’ consensus on the definition, diagnosis, and prevention of muscle diseases, considering different geographical features (2, 3). The second most-cited paper, “Sarcopenia, “was published in The Lancet in 2019. It summarizes the definition, screening, diagnosis, differential diagnosis, epidemiology, and pathophysiological mechanisms of sarcopenia treatment methods, including drug and non-drug therapies (23). Figure 10 displays the top 25 most cited references exhibiting the highest citation bursts, which initially emerged in 2004, with most occurring between 2012 and 2022, including 17 of the 25 peaks since the turn of the century. This indicates that the research in this field has been further developed and improved since 2012. In addition, due to the lag of literature citation, we can speculate that this field still receives continuous attention to this day.

**Figure 11:**
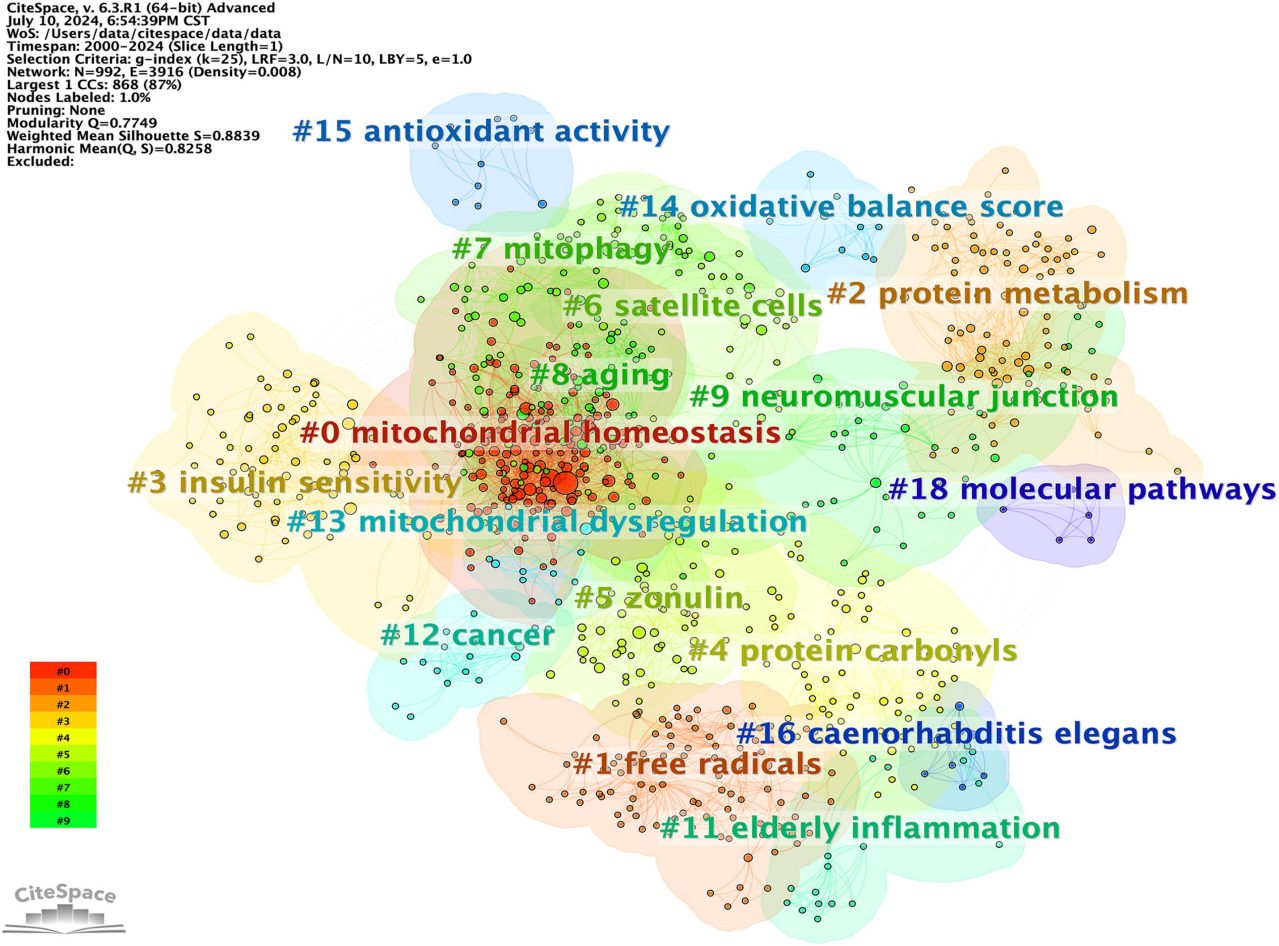
CiteSpace visualization co-cited references clusters map of OS in sarcopenia.

The subsequent step involved cluster analysis, as depicted in Figure 11. A knowledge graph was constructed from all co-cited references, comprising 19 clusters with distinct labels, providing an intuitive depiction of the temporal evolution of the oxidative stress (OS) in the sarcopenia research knowledge base (Table 6). It is noteworthy that the modularity value (Q-value) and mean silhouette value (S-value) are two key metrics used to evaluate the significance of community structure., with clustering being deemed significant when Q > 0.3 and S > 0.7 (24). Different clusters were obtained using the LLR clustering method. The Q-value (0.7749) and weighted mean silhouette (0.8839) indicated that the network was reasonable, suggesting that the co-cited references in each cluster were highly consistent. Based on the results of the clustering analysis, “mitochondrial homeostasis” (Cluster #0) emerged as the predominant cluster in this field, with an average publication year of 2018. The average year of co-cited reference literature for the most recent cluster was 2021, featuring mitochondrial dysregulation (Cluster #13), oxidative balance score (Cluster #14), and antioxidant activity (Cluster #15).

**Table 6:** Detailed information for co-cited references clusters.

ID	Size	Silhouette	Mean (year)	Label (LLR algorithm)
0	167	0.73	2018	Mitochondrial homeostasis
1	98	0.957	1998	Free radicals
2	91	0.919	2005	Protein metabolism
3	80	0.862	2013	Insulin sensitivity
4	76	0.859	2003	Protein carbonyls
5	66	0.929	2019	Zonulin
6	58	0.860	2011	Satellite cells
7	53	0.981	2011	Mitophagy
8	48	0.910	2015	Aging
9	41	0.919	2009	Neuromuscular junction
11	20	0.98	2003	Elderly inflammation
12	17	0.992	2020	Cancer
13	11	0.982	2021	Mitochondrial dysregulation
14	10	0.997	2021	Oxidative balance score
15	10	0.986	2021	Antioxidant activity
16	7	0.999	2003	*Caenorhabditis elegans*
18	6	1	2011	Molecular pathways
22	5	1	2018	Satellite cell
23	4	1	2019	Advanced glycation end product

### 3.6 Keyword co-occurrence and research clusters

**Figure 12:**
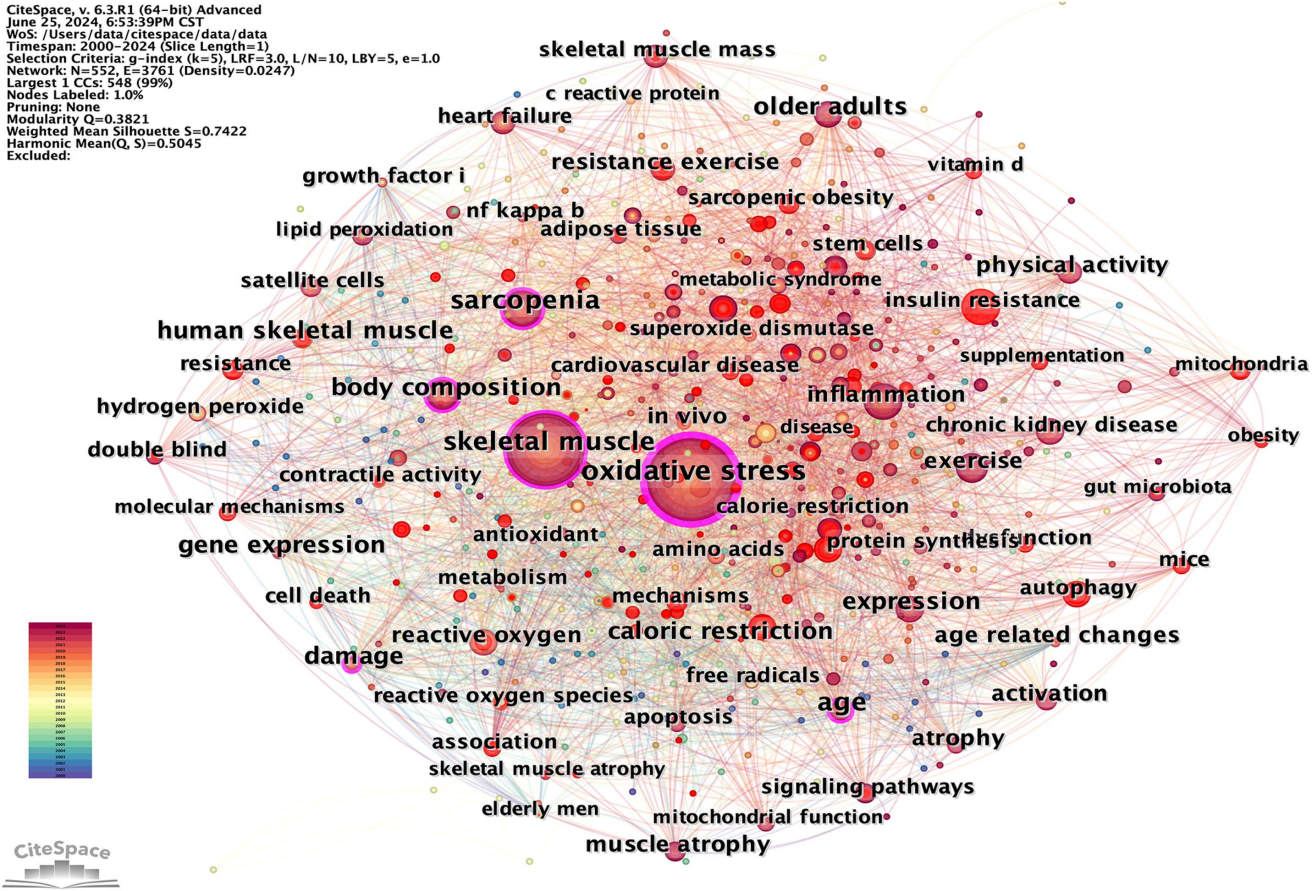
CiteSpace visualization co-occurring keywords map of OS in sarcopenia.

Keywords provide a summary of the abstract, and those with high frequency and centrality often indicate current hot topics in the field of research (25). Hot topics in the study can be revealed by clustering analysis of co-occurring keywords using CiteSpace. The merged co-occurring keywords network, depicted in Figure 12, consists of 552 nodes and 3,761 links. Based on frequency, the two most common keywords are “oxidative stress” and “skeletal muscle,” the themes of this study, pursued by “sarcopenia,” “exercise,” “expression,” “age,” “inflammation,” “body composition,” “older adults,” and “insulin resistance” (Table 7).

**Table 7:** Top 10 keyword with the highest counts.

Rank	Key word	Year	Counts	Centrality
1	Oxidative stress	2002	357	0.2
2	Skeletal muscle	2001	252	0.2
3	Sarcopenia	2009	103	0.11
4	Exercise	2004	68	0.05
5	Expression	2003	65	0.09
6	Age	2001	58	0.15
7	Inflammation	2008	58	0.03
8	Body composition	2003	57	0.1
9	Older adults	2012	55	0.06
10	Insulin resistance	2015	53	0.02

The 86 popular keywords appearing more than 10 times were divided into eight themes to determine the research focus (Table 8): Physiology and Anatomy Physiological Mechanism Pathology Associated Experimental Study Nutrition and Metabolism Sport and Physical Activity Age and Oxidation and Antioxidation

**Table 8:** Research keywords listed by theme.

Theme	Most frequent keywords
Physiology and Anatomy (12)	Skeletal muscle (252), human skeletal muscle (34), skeletal muscle mass (26), mass (26), muscle (23), muscle mass (23), muscle strength (10), stem cells (16), satellite cells (25), adipose tissue (23), mitochondria (14), gut microbiota (13)
Physiological Mechanism (16)	Oxidative stress (357), inflammation (58), autophagy (31), apoptosis (29), gene expression (29), protein synthesis (21), mitochondrial dysfunction (26), mitochondrial function (20), mitochondrial biogenesis (12), differentiation (11), regeneration (11), cell death (11), signaling pathways (13), molecular mechanisms (13), pathways (12), cellular senescence (10)
Pathology Associated (18)	Sarcopenia (103), muscle atrophy (39), atrophy (38), chronic kidney disease (28), insulin resistance (53), cardiovascular disease (18), skeletal muscle atrophy (17), sarcopenic obesity (20), heart failure (15), alzheimers disease (13), metabolic syndrome (12), disease (13), frailty (10), dysfunction (21), obesity (10), Health (17), stress (14), damage (31)
Experimental Study (10)	Expression (65), activation (44), double blind (13), association (21), risk (19), mechanisms (29), *in vivo* (22), mouse model (10), cells (18), mice (19)
Nutrition and Metabolic (8)	Body composition (57), protein (27), amino acids (15), supplementation (15), vitamin d (17), caloric restriction (23), calorie restriction (13), metabolism (23)
Sport and Physical Activity (7)	Exercise (68), physical activity (42), resistance exercise (27), contractile activity (12), physical function (13), strength (31), resistance (15)
Age (6)	Older adults (55), age (58), age related changes (21), life span (11), mortality (15), growth (10)
Oxidation and Antioxidation (9)	Reactive oxygen (32), nf kappa b (21), superoxide dismutase (20), reactive oxygen species (13), hydrogen peroxide (12), lipid peroxidation (14), nitric oxide (11), free radicals (11), c reactive protein (10)

**Figure 13:**
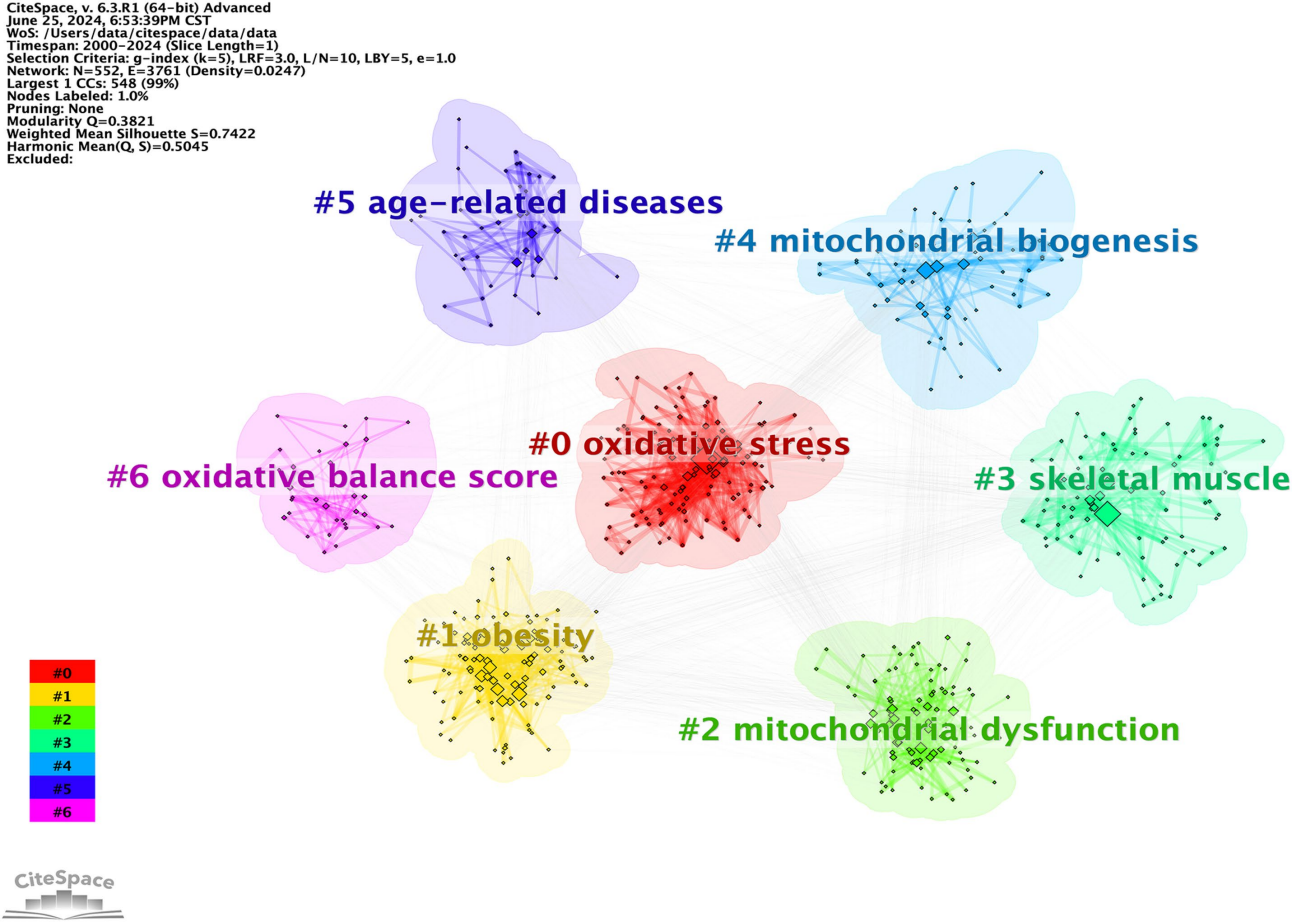
CiteSpace visualization co-occurring keywords clusters map of OS in sarcopenia.

Keyword clustering is a method of organizing and categorizing the internal knowledge structure of a particular research field, as illustrated in Figure 13 (26). Seven different clusters were obtained using the LLR clustering method. The Q-value (0.3821) and weighted mean silhouette (0.7422) indicated that the network was reasonable, suggesting that the keywords in each cluster were highly consistent. The average year of the keyword cluster corresponds to the mean publication year of the literature that encompasses these keywords (Table 9). The terms “obesity,” “mitochondrial dysfunction,” and “oxidative balance score” have emerged as prominent research areas in recent years. This indicates the research direction of the current stage.

**Table 9:** Detailed information for keywords clusters.

ID	Size	Silhouette	Mean (year)	Notation*
0	121	0.768	2007	oxidative stress
1	117	0.666	2016	obesity
2	91	0.677	2017	mitochondrial dysfunction
3	84	0.805	2008	skeletal muscle
4	61	0.752	2010	mitochondrial biogenesis
5	41	0.840	2012	age-related diseases
6	33	0.802	2015	oxidative balance score

*Notations were obtained via Log Likelihood Ratio (LLR) method.

### 3.7 Analysis of burst keywords

**Figure 14:**
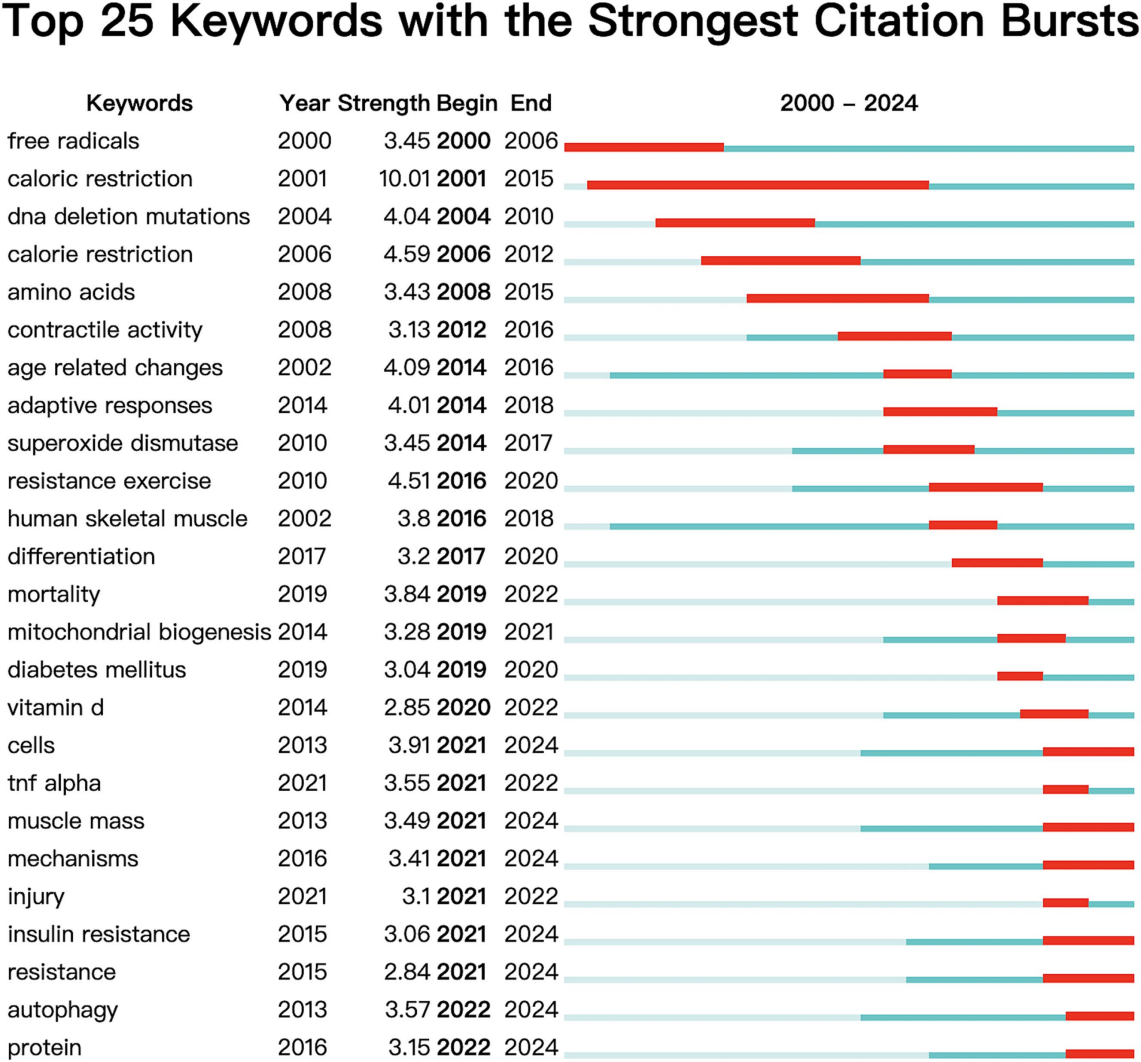
The top 25 keywords with the strongest citation burst.

Figure 14 displays the top 25 keywords exhibiting the most pronounced bursts. Blue bars denote the time interval, while red bars indicate the duration of each burst. It is evident that hot topics have shifted significantly. Since 2019, thirteen keywords, including “mortality,” “mitochondrial biogenesis,” “diabetes mellitus,” “vitamin D,” “cells,” “TNF-*α*,” “muscle mass,” “mechanisms,” “injury,” “insulin resistance,” “resistance,” “autophagy,” and “protein,” have begun to emerge prominently. Currently, seven terms continue to feature prominently in research: cells, muscle mass, mechanisms, insulin resistance, resistance, autophagy, and protein. This suggests that research on sarcopenia is at the forefront of OS, exploring novel complex cellular and molecular mechanisms of insulin resistance and muscle-related diseases.

## 4 Discussion

This study employed bibliometrics and visual analysis techniques to investigate the research hotspots and trends pertaining to OS in sarcopenia since the 21st century. The use of bibliometrics and visual analysis to discover international focal points and trends in this research. Domain provides valuable insights into evolving research patterns, helping researchers select future areas of investigation and appropriate journals.

### 4.1 Knowledge base

Based on the analysis, the quantity of published papers in this area has exhibited an upward trend since 2000. The number of published papers in 2023 was 4.5 times greater than in 2013 and 21.25 times greater than in 2003. The significant increase in publications indicates that OS in sarcopenia is gaining sustained attention from scholars worldwide as an emerging research focus.

Cooperative partnerships among nations, organizations, and scholars. The most impactful countries, institutions, and authors in the field of muscle symptoms were visually analyzed. Among the countries/regions with the greatest number of articles, North America and Eastern Asia have made significant contributions, while African countries have made the least. Articles from the United States, China, and Italy accounted for 56.94% of the total. The United States leads in terms of publications and intermediate centrality. Additionally, six European and North American countries—Italy, France, the United Kingdom, Spain, Canada, and Switzerland—are also in the top 10 for number of publications and intermediary centrality. In Asia, only China, South Korea, and the United Arab Emirates ranked among the top 10 in publication volume and intermediary centrality. South Korea (8) and the United Arab Emirates (10) ranked relatively low in intermediary centrality. This may be due to higher levels of scientific and technological advancement, earlier development, and greater recognition in Western countries. The institution with the greatest publication count in the institutional cooperation network is INRAE in France, while the State University System of Florida in the United States has the highest intermediary centrality. This indicates its pivotal role in linking institutions.

The findings of this study indicate that articles published in age-related and fundamental research journals tend to have higher impact factors, suggesting a greater level of quality. Additionally, Experimental Gerontology had the largest number of publications, and the Journals of Gerontology had the highest citation ranking. This shows that research in elderly diseases is taken seriously and that there is significant attention to basic research in this field. Holly Van Remmen from the Oklahoma Medical Research Foundation is a prominent scholar who has made significant contributions to the field. His extensive research focuses on investigating the mechanisms of OS in sarcopenia, including both upstream and downstream effects, as well as potential treatment interventions (27–29). In general, authors who are frequently cited are regarded as having greater influence than those who are cited less frequently, and co-cited authors often concentrate on related research areas. The scholar with the most citations is Cruz-Jentoft AJ from the Hospital Universitario Ramon y Cajal (IRYCIS) in Madrid, Spain. His primary research focus revolves around clinical aspects, specifically the conduction of clinical trials to investigate the causes and impacts on mortality of muscle diseases. Furthermore, he collaborates with other industry experts to publish expert consensus statements, which exert a significant influence in the field (30–32).

Among the top 10 cited documents, four are review articles, four original research articles, and two expert consensus statements. Interest in these articles has focused on sarcopenia screening and pathophysiological mechanisms, including inflammation, mitochondrial dysfunction, the coupling between increased OS and the disruption of cellular excitatory contraction mechanisms, as well as molecular reciprocity between muscle protein metabolism and therapeutic targets. Additionally, four original articles are dedicated to exploring and elucidating the intrinsic correlation between oxidative stress and sarcopenia. These articles explore various facets, encompassing physiological mechanism of muscle weakness caused by oxidative stress-induced excitation-contraction coupling disorder, the based part of mitochondrial metabolism alterations in elderly sarcopenia, and the involvement of Nrf2 in sarcopenia development. While citation analysis may not be as prompt as keyword analysis in identifying emerging frontiers, it is well-suited for elucidating the evolution of research fields. Therefore, recent clustering effectively showcases current trending topics and future directions (33). The average year of co-cited reference literature for the most recent cluster is 2021, highlighting mitochondrial dysregulation (cluster #13), oxidative balance score (cluster #14), and antioxidant activity (cluster #15). These areas warrant attention from researchers in future studies.

The term “keyword burst” refers to keywords extensively referenced in academic publications, serving as valuable indicators for identifying emerging trends and research hotspots within a specific field. Visualizing these keywords in Figure 14 helps us better understand the research hotspots and frontiers. The most frequent keywords are “oxidative stress” and “skeletal muscle,” which are the themes of this study. The analysis of 86 prominent keywords revealed that “Physiological mechanism” and “Pathology associated” emerged as the most prevalent terms. In addition to oxidative stress (357), sarcopenia (103), and muscle atrophy (39), which are the subjects of this study, it is noteworthy that inflammation (58), insulin resistance (53), and autophagy (31) are the three keywords with the highest frequency. Additionally, “TNF-*α* (inflammation), ““insulin resistance, “and “autophagy” were identified in the analysis of burst keywords, with chronologically ordered bursts, particularly emphasizing the persistence of the latter two keywords until the present time.

### 4.2 Research hotspots and frontiers

#### 4.2.1 Inflammation

A growing body of research has shown the crucial role of chronic inflammation in the development and advancement of sarcopenia. Inflammatory factors, particularly TNF-*α*, are believed to have a negative impact on muscle catabolism directly and indirectly. It has been noted that the release of TNF-*α* may adversely impact muscle strength and mass as sarcopenia progresses in elderly individuals. For instance, Li’s research indicates that elevated levels of TNF-α (>11.15 pg./mL) are associated with a 7.6-fold increase in the risk of sarcopenia compared to the control group (nonsarcopenia) (34). Consistent with the murine model, 24-month-old wild-type mice showed increased TNF-α levels and reduced muscle mass in the gastrocnemius, soleus, and quadriceps muscles compared to their 6-month-old counterparts (35).

Exposure to TNF-*α* leads to a significant loss of muscle mass and strength, closely connected with increased ROS production (36). Myostatin (Mstn), a negative regulator of muscle growth, influences the proliferation and differentiation of muscle cells and contributes to sarcopenia. Mstn acts as an oxidant by activating NF-κB and NADPH oxidase, which generates ROS in skeletal muscle cells, thereby inducing OS. TNF-*α* and hydrogen peroxide (H_2_O_2_) are potent inducers of Mstn expression, requiring NF-κB signal transduction for activation. A positive feedback loop exists between Mstn and TNF-α: Mstn triggers ROS production through TNF-α and NADPH oxidase, while elevated TNF-α levels stimulate Mstn expression. Elevated Mstn levels subsequently lead to muscle atrophy by promoting proteasome-mediated intracellular protein degradation (37).

Moreover, the study revealed that aging in mice resulted in a reduction of intestinal barrier thickness, leading to increased bacterial permeability and heightened release of damage-associated molecular patterns (DAMPs) (38, 39). Pattern recognition receptors recognize these DAMPs and trigger immune responses, resulting in elevated inflammation and the production of inflammatory cytokines such as TNF-*α* and IL-6 (40). Consequently, it is unsurprising that the gut microbiome can impact muscle function. TNF-α enhances the degradation of muscle proteins by activating the ubiquitin-proteasome pathway and upregulating atrogin1/MAFbx, a pivotal ubiquitin ligase involved in muscle breakdown (41). The role of TNF-α in muscle wastage has been confirmed through its complex signaling pathways, including cell death signal transduction. For instance, studies have indicated that targeting the Caspase-8/Caspase-3/GSDME signal-mediated cell pyroptosis pathway may hold promise as a therapeutic strategy for muscle-wasting disorders (42).

Recent investigations have initiated the exploration of TNF-α as a potential biomarker for sarcopenia. The assessment of TNF-α levels in blood or muscle tissue holds promise for the early prediction and diagnosis of sarcopenia (43–45). Therapeutic interventions targeting TNF-α, such as the administration of TNF-α inhibitors (e.g., etanercept and adalimumab), have demonstrated efficacy in various inflammatory conditions (46, 47). Eriodictyol, which naturally occurs in various fruits and vegetables, can activate the PI3K/AKT signaling pathway to alleviate oxidative stress associated with acute liver injury, but it has not been reported in sarcopenia related studies (48). Ongoing research is investigating the potential utility of these agents in mitigating or delaying the progression of sarcopenia. Etanercept has the potential to attenuate fibrotic transformation, reduce muscle wasting, enhance muscle function, and consequently extend the lifespan of aging mice (49, 50). However, in light of the current state of research, further refinement is required for the clinical application of anti-inflammatory cytokine therapy in sarcopenia. This includes addressing late-stage disease treatment and determining the optimal dosage to effectively inhibit inflammatory processes, irrespective of concurrent drug usage.

#### 4.2.2 Insulin resistance

Insulin resistance (IR) is characterized by decreased sensitivity of the body to insulin, resulting in abnormal glucose metabolism. It is highly prevalent in clinical settings and is considered a common underlying factor for chronic metabolic disorders and the pathophysiological basis for certain rare diseases. According to the National Health and Nutrition Examination Survey in the United States, approximately 40% of adults aged 18 to 44 exhibit IR (51). Age-related insulin resistance is related to impaired muscle glucose metabolism, which results in compromised cellular energy production and weakened muscle contraction. Furthermore, a detrimental feedback loop exists between insulin function and age-related decline, worsening insulin resistance (52, 53). The association between muscle characteristics and insulin resistance is strongest in terms of relative muscle mass (54).

With aging, the muscle cell composition shifts from type II fast fibers to type I slow fibers, which have reduced aerobic respiration capacity and a diminished ability to perform rapid, forceful movements. Additionally, muscle quality declines due to adipose tissue infiltration within the muscles, including both intramuscular and intermuscular fat. This infiltration correlates significantly with insulin resistance and is an independent predictor of decreased physical function and increased risk of falls (3). On one hand, insulin resistance can impede the PI3K/protein kinase B signaling pathway, thereby suppressing protein synthesis and reducing muscle growth. On the other hand, insulin resistance may hinder the translocation of glucose transporter 4, thereby reducing muscle glycogen synthesis and resulting in an inadequate energy supply for muscles and decreased muscular strength. Furthermore, insulin resistance has the potential to induce cellular autophagy and mitochondrial dysfunction, which consequently impacts muscular strength (55).

The mammalian target of rapamycin (mTOR) pathway is crucial for muscle synthesis, whereas muscle breakdown is primarily regulated by the ubiquitin-proteasome pathway. IR leads to an imbalance between these processes (56, 57). Meanwhile, elevated levels of TNF-*α* have been demonstrated to inhibit the AKT/mTOR pathway, governing skeletal muscle hypertrophy, thereby enhancing muscle breakdown (58). However, Sasako et al. showed that interfering with the insulin/IGF-1 signaling pathway by suppressing Akt activity in mouse skeletal muscle can accelerate sarcopenia and reduce lifespan, a phenomenon reversed by deactivating FoxOs rather than activating mTOR (59). Moreover, inflammatory factors (such as TNF-α, IL-1β, and IFN-*γ*) can further exacerbate IR, resulting in increased inflammation and OS. This establishes a detrimental cycle of muscle wasting, IR, inflammation, and OS, contributing to a decline in muscle mass and a rise in fatness (60, 61). In conclusion, decreased skeletal muscle mass may hinder insulin-driven glucose disposal due to its role as the primary tissue governing insulin-regulated glucose metabolism. This, in conjunction with increased insulin resistance during skeletal muscle aging, contributes to the cascades driving disease progression (62, 63).

The management strategies for IR encompass two key components: lifestyle interventions and pharmacotherapy. Lifestyle interventions include adherence to a nutritious diet, increased physical activity, stress reduction, smoking cessation, alcohol moderation, regular sleep patterns, and supplementation with essential minerals and trace elements. Notably, Harvie et al. observed that the 5: 2 dietary pattern, compared to mere calorie control, demonstrates greater efficacy in ameliorating insulin resistance in the short term (64). Although exercise can enhance insulin sensitivity, the superiority of anaerobic exercise over aerobic exercise in improving insulin resistance remains inconclusive (65). Chobanyan-Jurgens et al. reported that hypoxic endurance training does not confer a superior improvement in insulin resistance compared to normoxic training (66). Conversely, Mai et al. demonstrated that hypoxic exercise results in greater enhancement of insulin resistance compared to normoxic exercise (67). In summary, both moderate aerobic exercise and anaerobic exercise are beneficial for individuals with sarcopenia. At present, there are no drugs sanctioned by regulatory authorities treating muscle wasting related to insulin resistance (68). Presently, therapeutic indications for addressing insulin resistance primarily pertain to type 2 diabetes mellitus, making drug therapy generally appropriate for individuals with both conditions. In cases where insulin resistance coexists with sarcopenia in non-diabetic patients, viable symptomatic treatment alternatives are lacking when lifestyle interventions fail to adequately manage both conditions. Significant advancements are anticipated in this domain.

#### 4.2.3 Autophagy

Autophagy is a highly conserved, lysosome-dependent degradative metabolic process wherein cellular components, including damaged organelles, protein aggregates, and lipid droplets, undergo degradation and subsequent recycling (69). The classical autophagy process encompasses five stages: phagophore formation, nucleation, elongation, autophagosome-lysosome fusion, and degradation (70). Autophagy is facilitated in the elimination of damaged mitochondria and proteins from muscle cells, thereby preserving their health and functionality. Specifically, moderate autophagy can mitigate the accumulation of detrimental substances, diminish OS and inflammation within cells, consequently upholding muscle quality (71, 72). However, exorbitant autophagy may cause the heightened degradation of muscle proteins, thereby exacerbating muscle atrophy and loss of function. In certain pathological conditions, the dysregulation of autophagy, such as overactivation or insufficiency, may serve as a pivotal driving force in sarcopenia (73, 74).

Research has demonstrated that impaired autophagy disrupts cellular homeostasis, impairs mitochondrial function, intensifies OS, and accelerates cellular senescence, thereby compromising satellite cell (75–77). Satellite cells reside between the plasma membrane and the basal lamina of muscle fibers. They have demonstrated effectiveness and autonomy in muscle regeneration, capable of proliferating and differentiating into various types of muscle fibers in response to mechanical damage in the extracellular environment (78, 79). A decrease in satellite cells, or a decline in their function, can compromise muscle regeneration, ultimately resulting in sarcopenia (80, 81). Researchers discovered that activation of the autophagy-AMPK/p27(Kip1) pathway enhances the proliferation efficiency of aging satellite cells, mitigating cell apoptosis and senescence, and ultimately improving muscle regeneration (82). SIRT1 may activate autophagy via the FoXO3a way to satisfy the bioenergetic demands of stimulating satellite cells (83, 84). Disruption of autophagy in satellite cells through ATG7 knockout may impede their regeneration by downregulating the levels of growth hormone receptor (GHR) and insulin-like growth factor-1 (IGF-1) (85). IGF-1 is crucial in different anabolic metabolic pathways within skeletal muscle. Studies suggest that the IGF-1Ea and IGF-1Eb subtypes can induce autophagy/lysosome system activation, which is generally modified in the aging process. Furthermore, these subtypes promote the upregulation of PGC1-*α* expression, thus affecting mitochondrial function, enhancing ROS detoxification, and adjusting the inflammatory situation in elderly participants (86).

Autophagy plays a crucial role in clearing excessive ROS generated during OS. Studies have shown that compromised autophagic function leads to ineffective regulation of pathological OS within cells (75, 87, 88). Autophagy competes with p62 for binding to Nrf2, inhibiting its degradation and mitigating OS responses (89). Nrf2 depletion enhances the OS response and increases ROS levels by modulating the expression of antioxidant genes (90, 91). Additionally, autophagy can inhibit OS and degrade NLRP3 to prevent the generation of the inflammatory factor IL-1β (92). When NLRP3 is activated, impaired autophagy results in elevated IL-1β expression, which triggers the NF-κB signaling pathway, exacerbating the onset of sarcopenia (93, 94).

Exercise-induced autophagy is promoted through the Akt/mTORC or FoxO3a pathways, potentially representing one of the mechanisms by which aerobic and resistance exercises mitigate muscle loss (95, 96). When long-term supplementation with spermidine, an autophagy inducer, activates autophagy, satellite cell proliferation is increased (97). While the role of spermine in enhancing mitophagy to mitigate oxidative stress-induced cardiac damage is well-established, there is a lack of research exploring its potential beneficial effects on sarcopenia (98, 99). Melatonin, an antioxidant, can promote mitophagy via the cGAS-STING-TBK1 pathway, thereby ameliorating oxidative stress and inflammatory responses. However, the clinical efficacy of melatonin in the treatment of sarcopenia remains uncertain (100, 101).

### 4.3 Strengths and limitations

The study is constrained by the limited availability of data sources and analytical tools. The primary data source used was the WOS Core Collection database, which may result in the omission of relevant literature. In addition, The WOS database has known biases toward English-language publications and certain geographic regions, potentially underrepresenting valuable research from non-English speaking countries. While CiteSpace software is beneficial for bibliometric analysis, its algorithms and visualization outcomes can be affected by the quantity and quality of the literature analyzed. Additionally, the criteria for selecting literature and the methods for data processing may introduce bias, as no retrieval strategy is flawless. To achieve the most reliable and relevant results, we focused on gathering high-quality literature. What is more, the analysis does not account for negative citations or self-citations, which may inflate impact metrics. On top of that, the real-time updating of the online database introduces a time-sensitive element to bibliometric analysis. Despite these challenges, this study has encompassed the majority of published research on OS in sarcopenia since the 21st century, effectively capturing the prevailing research focal points, developmental trajectory, and emerging trends in this domain. Future studies should consider integrating multiple databases and employing diverse analytical tools for cross-validation.

## 5 Conclusion

This study marks the pioneering application of bibliometric and visualization analysis in the field of OS in sarcopenia. In this field it has also carried on the comprehensive analysis of the pattern of global research, identified the key focus, summarized the research status and put forward the possible future research priorities. Since the early 21st century, there has been a steady rise in the number of publications, with great contributions from the United States, China, and Italy. Holly Van Remmen is the most prolific author in this field. Experimental Gerontology has the highest volume of published articles, whereas the Journal of Gerontology Series A: Biological Sciences and Medical Sciences holds the record for the highest number of citations. Future research is supposed to pay attention to “TNF-*α*,” “insulin resistance,” “mitochondrial autophagy,” “signaling pathways,” and “mechanisms” as pivotal areas in the study of oxidative stress in sarcopenia. The current literature review suggests that more high-quality basic research and clinical translational studies are necessary to determine the most effective strategies for reducing oxidative stress in sarcopenic patients, potentially improving their quality of life. Therefore, to benefit more patients with sarcopenia, enhanced collaboration and communication among institutions and research teams are essential for future advancements in this field.

## Data Availability

The raw data supporting the conclusions of this article will be made available by the authors, without undue reservation.
