# Supplementary material for: Scientific landscape of oxidative stress in sarcopenia: from bibliometric analysis to hotspots review
Source: Front Med (Lausanne). 2024 Nov 11;11:1472413. doi: 10.3389/fmed.2024.1472413 (PMC11586176; doi:10.3389/fmed.2024.1472413)
Supplement: Supplementary file 1 [file Table_1.DOCX]

Supplementary Material

# Supplementary Table

For more information on Supplementary Material and for details on the different file types accepted, please see [here](https://www.frontiersin.org/guidelines/author-guidelines#supplementary-material).

## Supplementary Figures

Table S1: Search terms related to oxidative stress and sarcopenia of this study.

| Search term |  |
| --- | --- |
| #1 | (TI=("Oxidative Stress*" OR "Antioxidative" OR "Anti-oxidative" OR "Oxidative Damage" OR "Oxidative Injury" OR "Oxidative and Nitrosative Stress" OR "Oxidative-Nitrosative Stress" OR "Reactive Oxygen Species" OR "ROS" OR "Nitrosative Stress" OR "Reactive Nitrogen Species" OR "RNS" OR "Free Radical*" OR "Catalase" OR "Superoxide Dismutase" OR "SOD" OR "Glutathione" OR "GPx" OR "NADPH oxidase*" OR "Nrf2" OR "Nuclear Factor E2 Related Factor 2" OR "Oxidant*" OR "Lipid Peroxidation" OR "Hydrogen Peroxide" OR "H_2_O_2_")) AND (TI =( "sarcopenia " OR "sarcopenic")) |
| #2 | (AK=("Oxidative Stress*" OR "Antioxidative" OR "Anti-oxidative" OR "Oxidative Damage" OR "Oxidative Injury" OR "Oxidative and Nitrosative Stress" OR "Oxidative-Nitrosative Stress" OR "Reactive Oxygen Species" OR "ROS" OR "Nitrosative Stress" OR "Reactive Nitrogen Species" OR "RNS" OR "Free Radical*" OR "Catalase" OR "Superoxide Dismutase" OR "SOD" OR "Glutathione" OR "GPx" OR "NADPH oxidase*" OR "Nrf2" OR "Nuclear Factor E2 Related Factor 2" OR "Oxidant*" OR "Lipid Peroxidation" OR "Hydrogen Peroxide" OR " H_2_O_2_")) AND (AK =( "sarcopenia " OR "sarcopenic")) |
| #3 | (AB=("Oxidative Stress*" OR "Antioxidative" OR "Anti-oxidative" OR "Oxidative Damage" OR "Oxidative Injury" OR "Oxidative and Nitrosative Stress" OR "Oxidative-Nitrosative Stress" OR "Reactive Oxygen Species" OR "ROS" OR "Nitrosative Stress" OR "Reactive Nitrogen Species" OR "RNS" OR "Free Radical*" OR "Catalase" OR "Superoxide Dismutase" OR "SOD" OR "Glutathione" OR "GPx" OR "NADPH oxidase*" OR "Nrf2" OR "Nuclear Factor E2 Related Factor 2" OR "Oxidant*" OR "Lipid Peroxidation" OR "Hydrogen Peroxide" OR " H_2_O_2_")) AND (AB=(( "sarcopenia " OR "sarcopenic"))) |
| #4 | PY = "2000-01-01~2024-06-23" |
| #5 | Article type="article" and "review" |
| #6 | (#1 OR #2 OR #3) AND #4 AND #5 |
